# Bandgap‐Coupled Template Autocatalysis toward the Growth of High‐Purity sp^2^ Nanocarbons

**DOI:** 10.1002/advs.202003078

**Published:** 2021-02-18

**Authors:** Jun Gao, Zhenxing Zhu, Boyuan Shen, Yunxiang Bai, Silei Sun, Fei Wei

**Affiliations:** ^1^ Beijing Key Laboratory of Green Chemical Reaction Engineering and Technology Department of Chemical Engineering Tsinghua University Beijing 100084 China

**Keywords:** autocatalysis, bandgap, carbon nanotube, defect, graphene

## Abstract

Extraordinary properties and great application potentials of carbon nanotubes (CNT) and graphene fundamentally rely on their large‐scale perfect sp^2^ structure. Particularly for high‐end applications, ultralow defect density and ultrahigh selectivity are prerequisites, for which metal‐catalyzed chemical vapor deposition (CVD) is the most promising approach. Due to their structure and peculiarity, CNTs and graphene can themselves provide growth templates and nonlocal dual conductance, serving as template autocatalysts with tunable bandgap during the CVD. However, current growth kinetics models all focus on the external factors and edges. Here, the growth kinetics of sp^2^ nanocarbons is elaborated from the perspective of template autocatalysis and holistic electronic structure. After reviewing current growth kinetics, various representative works involving CVD growth of different sp^2^ nanocarbons are analyzed, to reveal their bandgap‐coupled kinetics and resulting selective synthesis. Recent progress is then reviewed, which has demonstrated the interlocking between the atomic assembly rate and bandgap of CNTs, with an explicit volcano dependence whose peak would be determined by the environment. In addition, the topological protection for perfect sp^2^ structure and the defect‐induced perturbation for the interlocking are discussed. Finally, the prospects for the kinetic selective growth of perfect nanocarbons are proposed.

## Graphical abstract

The developments toward perfect nanocarbon structures are presented, and the importance of bandgap‐coupled template autocatalysis kinetics is emphatically demonstrated. The protection provided by large formation energies of topological defects for perfect sp^2^ structure is revealed. Furthermore, the bandgap‐coupled autocatalysis growth kinetics, which could modulate detailed structure of nanocarbons due to their slight difference in bandgap, are manifested by various evidences.

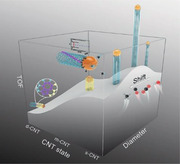

## 1 Introduction

Nanocarbons, appearing in many allotropes from 0D to 3D, have drawn sustained interest in the past several decades, among which carbon nanotube (CNT) and graphene are two representative sp^2^ nanocarbons with honeycomb lattice.^[^ ^1^, ^2^ ^]^ Due to their unique atomic network and accompanying novel properties, they have been investigated extensively, from structure, properties to synthesis and applications.^[^ ^3^, ^4^, ^5^, ^6^, ^7^, ^8^, ^9^, ^10^ ^]^ Perfect structure consistency in a vast range is fundamentally crucial for the properties and applications, having prompted the discovery of up‐to‐date strongest CNT fibers,^[^ ^11^ ^]^ the macroscale superlubricity of CNTs^[^ ^12^ ^]^ and superconductivity within crystalline of twisted graphene.^[^ ^13^ ^]^ Plenty of functional devices based on sp^2^ nanocarbon materials have successively emerged, from the minimum CNT transistors contacted with graphene electrodes^[^ ^14^ ^]^ to the CNT‐based computing and storage device,^[^ ^15^ ^]^ microprocessor,^[^ ^16^ ^]^ even computer.^[^ ^17^ ^]^ Achieving large‐scale production of desired materials is a prerequisite to implement these attempts into practical applications. It is extremely demanding in the high‐end applications of electronic and mechanical fields, where CNTs and graphene with defined structure and defect‐free characteristic are required, even an ultrahigh semiconducting CNT (s‐CNT) selectivity of 99.9999% and an ultralow defect density of 10^−12^ to 10^−13^ for integrated circuits and ultrastrength CNTs bundles.

Among various strategies for nanocarbons preparation, catalytic chemical vapor deposition (CVD) has become the most promising method, for its potential to achieve wafer‐scale, high‐quality, and controlled‐parameter synthesis. In CVD growth of nanocarbons, catalysts play a critical role in providing matched symmetry and proper interfacial energy with the infant nanocarbon templates. Some recent advances in kinetic reproduction and kinetic selective growth have attracted more attention to the breeding from infant nanocarbons. Compared with thermodynamic match,^[^ ^18^, ^19^ ^]^ growth kinetics are more significant in the evolution of infant nanocarbons into larger scale, dominating numerous aspects, such as the appropriate growth rate to determine the quality and duration of crystallization, the definite morphology to tune the bandgap and further device characteristics, and the prevention of topological defects.

To investigate the growth kinetics of nanocarbons, external parameters have already been focused on, like temperature, gas atmosphere, interacting field, etc., which were verified to be critical in controlling the carbonaceous dissolution, precipitation, and precise assembly into sp^2^ nanostructure. The effects of these parameters have been summarized as a synergistic influence on the catalyst activity probability,^[^ ^20^ ^]^ to address the exponential decay rate in chain growth. But still, little attention has been devoted to the internal factors. It remains controversial how the topological edges (combinations of armchair, AC or zigzag, ZZ sites) and the bandgap of infant graphene or CNTs affect their kinetic growth in catalytic CVD. The effect would be extremely prominent, considering the ability of sp^2^ nanocarbons serving as growth templates and effective catalysts themselves, the latter of which is called carbocatalysis and has been exemplified by numerous experimental achievements,^[^ ^21^, ^22^, ^23^ ^]^ such as in oxidative dehydrogenation^[^ ^24^ ^]^ and hydrogenation reactions.^[^ ^25^, ^26^ ^]^ There is still a lack of an explicit relationship among the edge, bandgap, carbocatalysis kinetics, and the perfect sp^2^ structure.

**Figure 1:**
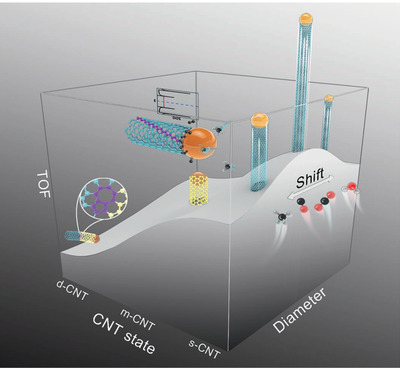
Schematic illustration of bandgap‐coupled growth kinetics of CNT. Perfect sp^2^ structure is protected by the large formation energy of topological defects, resulting in the relative lack of defective CNT (d‐CNT) under proper CVD conditions. Bandgap is significant for the kinetic growth of CNTs. The atomic assembly rate, depicted with turnover frequency (TOF), is prominently lower for metallic CNT (m‐CNT) than that for s‐CNT, and the latter manifests a volcano dependence on the bandgap that is inversely proportional to diameter, while the peak position of the volcano would be shiftable and determined by environment.

Herein, we would review the kinetic basis and progress for selective preparation of perfect nanocarbon structures, and emphatically demonstrate the important role of bandgap‐coupled template autocatalysis. First, we introduce the structure, bandgap, and existing growth kinetics of sp^2^ nanocarbons. Then more light would be shed on the significance of bandgap for the selective growth, to reveal the basis for the bandgap‐coupled template autocatalysis kinetics, which could modulate detailed structure of nanocarbons due to their difference in bandgap. Further, this unique kinetics would be exemplified by various evidences, including CVD growth of graphene nanoribbons and graphene, cloning CNT growth, environment‐determined bandgap distribution of CNTs and so on. Then we review our recent work in rate‐selected synthesis of perfect ultralong s‐CNTs, which manifested the interlocking between atomic assembly rate and bandgap of CNTs, with a volcano kinetics dependence on the bandgap (**Figure** **1**). Subsequently, the topological protection for perfect sp^2^ structure by high formation energy of defects and the disruptive impact of defects on the interlocking would be discussed. Finally, we summarize the challenges and prospects for future research in kinetic growth of perfect sp^2^ nanocarbons, in the hope of stimulating a fresh understanding toward controlled growth of perfect nanocarbons to achieve their high‐end applications.

## 2 Morphology and Band Structures of sp^2^ Nanocarbons

**Figure 2:**
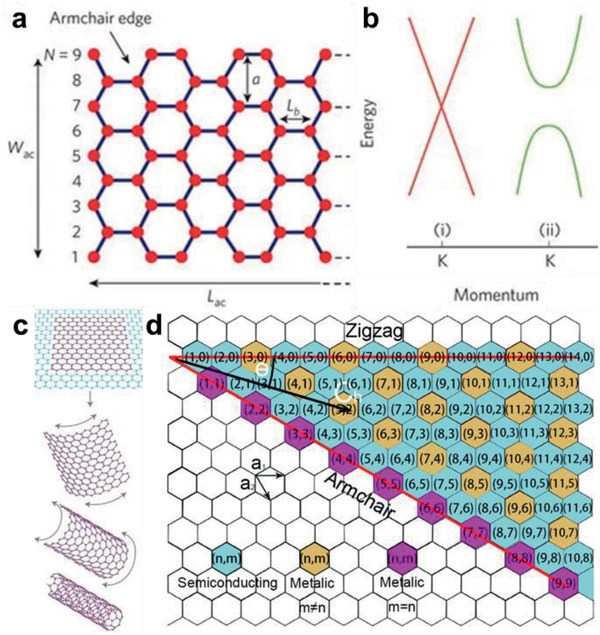
Structure of sp^2^ nanocarbons. a) Schematic illustration of an armchair graphene nanoribbon. b) Band structure around the K point of i) zero‐bandgap graphene, ii) graphene nanoribbons with an open bandgap. a,b) Adapted with permission.^[^ ^28^ ^]^ Copyright 2010, Springer Nature. c) Graphene can be rolled into CNT. Adapted with permission.^[^ ^3^ ^]^ Copyright 2007, Springer Nature. d) Chirality map of CNTs. The chiral vector *C*_h_ = *na*_1_+*ma*_2_ and chiral angle *θ* are displayed. And the species with *n* − *m* = 3*q*, *q* = 0 (*q* is an integer), *n* − *m* = 3*q*, *q* ≠ 0 and *n* − *m* = 3*q* ± 1 are labeled in purple, yellow, and green, respectively.

Graphene, one layer of carbon atoms closely packed in honeycomb lattice (**Figure** **2**a), is the 2D prototype for other sp^2^ hybridized nanocarbons. It is acknowledged that graphene possesses symmetric linear dispersion at the fermi energy level without bandgap.^[^ ^27^, ^28^ ^]^ But the bandgap would be opened up if the charge carriers are confined in quasi‐1D nanoscale (Figure 2b), which stimulates the formation of graphene nanoribbons (GNRs).^[^ ^29^, ^30^ ^]^ And the bandgap is inversely proportional to the width *w* of the GNR, following the relationship *E*_g_ ≈ *α*/*w*, where *α* is a fitted constant.^[^ ^31^, ^32^, ^33^, ^34^ ^]^ Edges play an important role in graphene nanostructures, which are composed of fundamental zigzag/armchair edges, and arbitrary edges with a slanted angle from the zigzag edge.

CNT can be viewed as a coaxial cylinder rolled up from graphene along a chiral vector *C*_h_ = *n* ***a*_1_** + *m* ***a*_2_**, where *n*, *m* are integers called chiral index and ***a*_1_**, ***a*_2_** are the unit vectors of the graphene lattice (Figure 2c,d). The width of graphene layer and the rolling up direction would determine the diameter *d* and chiral angle *θ* of CNT, which can be embodied in chiral index. The geometry of CNT edge and spiral chain can thus be determined with chiral index and the electronic structures can be corroborated as well. It is well established that metallic CNTs would form when *n* − *m* = 3*q* (*q* is an integer), where the electronic bands cross at the Fermi level, but due to curvature effect, a tiny bandgap opens up when *q* ≠ 0, which follows the relationship *E*_g_ ≈ 1/*d* ^2^.^[^ ^35^ ^]^ When *n* − *m* = 3*q* ± 1, they are semiconducting and bandgap is roughly inversely proportional to diameter, depicted as *E*_g_ ≈ 2*a*_C–C_ *γ*_0_/*d*, where *a*_C–C_ is the nearest‐neighbor C–C distance, *γ*_0_ is the nearest‐neighbor C–C interaction energy.^[^ ^36^, ^37^ ^]^

## 3 Edge‐Controlled Catalytic Growth Kinetics

In the CVD growth of sp^2^ nanocarbons, edges would act as the templates and active sites where sustained atomic assembly occurs. The interaction between edges and metal catalysts is a key issue to influence the activation energy, also concerned about the growth of perfect large‐size nanocarbons.^[^ ^38^ ^]^ Furthermore, the energy difference among zigzag, armchair edge, or their combination would not only impact the structure control, but also influence the growth kinetics.

### 3.1 Kink‐Determined Growth Kinetics for Graphene

**Figure 3:**
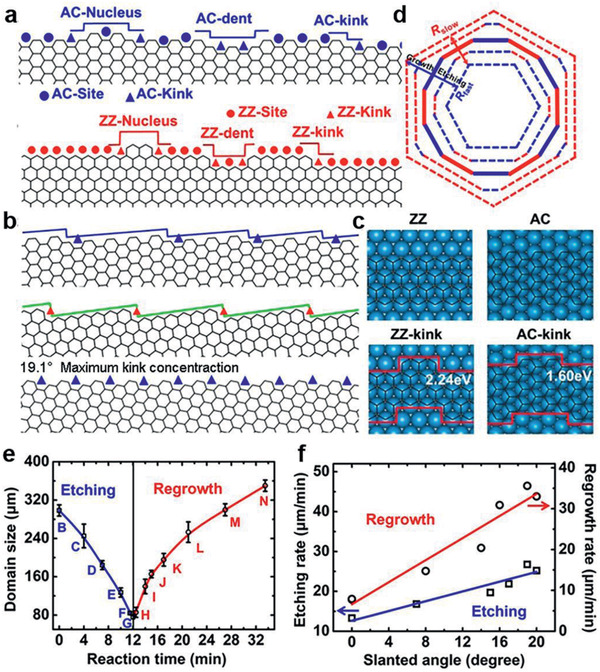
Kink‐determined growth kinetics for graphene. a) Nucleus, dent, and kink formation on the AC and ZZ edges. b) From up to bottom: slanted edges viewed as an AC and a ZZ edge with a quantity of kinks, and that with 19.1° tilt angle exhibiting the highest concentration of kinks. c) The formation energies of the kinks on the ZZ and AC edges on the Pt(111) surface. d) Illustration of the graphene shape change during growth (the outward arrow) and etching (the inward arrow). e) Time evolution of the graphene domain size during etching and regrowth. The errors come from the variation in size. f) The rates of etching and regrowth of the graphene versus the average slanted angle ****χ****. The red and blue lines represent the linear fits. a–f) Adapted with permission.^[^ ^39^ ^]^ Copyright 2013, National Academy of Sciences.

As an elementary step of graphene growth, carbon accretion onto the edge is vital to the kinetics, which has been demonstrated by theoretical and experimental investigations in the growth, etching, and regrowth processes.^[^ ^39^ ^]^ Taking the growth process as an example, calculations have explicitly revealed that the nucleus on a ZZ edge possesses higher formation energy than that on an AC edge (**Figure** **3**c), resulting in a slower growth rate of a zigzag edge than an armchair edge. Other edges can be considered as an armchair or zigzag edge with kinks, the most active sites for carbon accretion (Figure 3a,b). Consequently, the growth rate of a slanted edge is linearly proportional to the concentration of kinks, the maximum of which exists in the edge with a 19.1° tilt angle from the zigzag direction. This trend also fits well with the etching rate.

In brief, the theoretical growth/etching rates of different graphene edges follow the sequence: *R*_ZZ_ < *R*_AC_< *R*_tilt_ < *R*_19.1°_ where *R*_tilt_ is the rate of edges with medium tilt angles. As Figure 3d–f manifest, this kinetics model has also been demonstrated by experimental results of the graphene growth/etching process.

### 3.2 Screw Dislocation Theory for CNTs and Its Development

**Figure 4:**
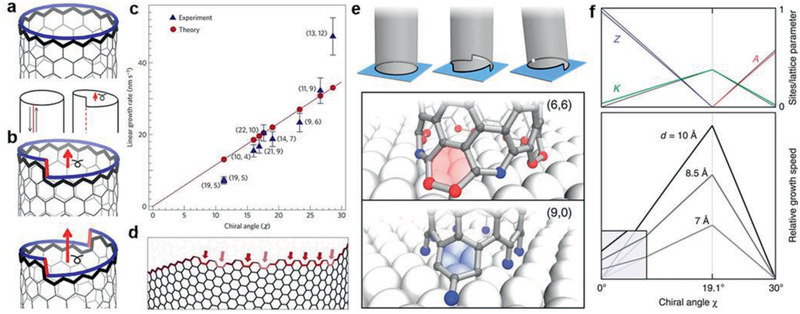
Edge‐determined growth kinetics for CNTs. a) An achiral zigzag (*n*, 0) tube that can be viewed as a perfect crystal, and how it transforms into a chiral one. b) (*n*,1) and (*n*,2) CNTs containing one and two kinks, respectively. The kinks at the edge are marked in red. a, b) Reproduced with permission.^[^ ^40^ ^]^ Copyright 2009, National Academy of Sciences. c) The experimental results to confirm the screw dislocation model. The blue and red labels represent growth rates of different CNTs from experiment and theory, respectively. d) A schematic illustration of different site types on CNT edges. Different colors indicate highly active kinks (light red), moderately active armchair(red) and inactive zigzag (dark red) sites for carbon addition. c,d) Reproduced with permission.^[^ ^41^ ^]^ Copyright 2012, Springer Nature. e) Schematic illustration of achiral, multiple‐kink chiral and single‐kink chiral CNTs on a flat substrate and the atomic configurations of (6,6), (9,0) tubes after incorporating first dimer. f) The density plot of different site types on CNT edges and the resulting growth rates with different diameters, as a function of chiral angle. e,f) Reproduced with permission.^[^ ^42^ ^]^ Copyright 2014, Springer Nature.

In terms of the CNTs, the incorporation of carbon atoms onto the open ends is equivalently important. Ding et al.^[^ ^40^ ^]^ proposed that during CNT growth, carbon dimers accumulate onto the CNT rim in a screw dislocation way. In the theory based on vapor–liquid–solid mode, a chiral (*n*, *m*) CNT can be viewed as a defective crystal derived from an achiral zigzag CNT, with a quantity of axial screw dislocations and resultant kinks (**Figure** **4**a,b). There are *m* kinks on the end‐rim of a (*n*, *m*) CNT with the growth rate of *k*_0_, so that the total carbon deposition rate can be estimated as *K* = *k*_0_ × *m*.

It means that the growth rate is proportional to the concentration of armchair kinks and chiral angle, while armchair (*n*, *n*) CNTs with 30° chiral angle possess the fastest growth rate. Experimentally, such a screw dislocation theory was further confirmed by Maruyama and co‐workers^[^ ^41^ ^]^ using in situ Raman spectroscopy. They measured growth rate and assigned chiral indices of nine single‐walled CNTs (SWNTs), obtaining a positive linear relationship between the growth rate and chiral angle, as displayed in Figure 4c,d.

Furthermore, Yakobson and co‐workers^[^ ^42^ ^]^ improved the model by considering the tube‐catalyst interface and the kinks created by thermal fluctuations on achiral edges. In their model, the CNT is in contact with an atomic terrace of the solid catalyst particle. As displayed in Figure 4e, it would destroy the perfect contact and cost noticeable energy to create a pair of kinks on the achiral edges, resulting in ultralow growth rates of zigzag and armchair CNTs. Ultimately, the growth rate in the whole chiral angle range can be derived to be nearly proportional to the density of geometric kinks, which would increase with the vicinal‐edge angular deviation from the armchair/zigzag achiral direction (Figure 4f). According to the model, for the growth of CNTs on solid catalyst, the fastest growth rate belongs to (2*m*, *m*) CNTs with 19.1°chiral angle, whose kink density at the edge is the largest.

The theory of screw dislocation has provided a robust foundation and instruction for chirality‐specific synthesis of CNTs. The active sites constructed by the kinks along the rim have clearly indicated the role of templates in differentiating the kinetic preference toward specific chiral CNT species.

## 4 Template Autocatalysis and Bandgap‐Coupled Growth Kinetics

Besides the edges, bandgap is another factor that is significant but often overlooked in the kinetic growth of sp^2^ nanocarbons. Unique properties endow nanocarbons with the autocatalysis ability, providing a link between the tunable bandgap of nanocarbons and their catalytic growth kinetics.

### 4.1 Basis for the Bandgap‐Coupled Template Growth Kinetics

In heterogeneous catalysis of transition metals, it is common to modulate the type and composition of metal catalysts to increase catalytic rates and efficiencies for different reactions, such as the reactions of C*_x_*H*_y_* and ammonia synthesis that can be up to industrial scale. The corresponding theoretical support has been revealed,^[^ ^43^, ^44^, ^45^ ^]^ in which a linear relation called Brønsted–Evans–Polanyi relation, is obeyed between transition‐state energy to adsorption (*E*‡) and the dissociative chemisorption energy (Δ*E*). For the situation that dissociative adsorption of a reactant is rate‐limiting, the overall reaction rate would behave as a volcano dependence versus Δ*E*, with a maximum rate in a specific intermediate range of Δ*E*.^[^ ^45^ ^]^ Furthermore, Δ*E* is related to the relative position of the d‐band center to the Fermi energy in transition metals,^[^ ^46^, ^47^, ^48^ ^]^ which can be determined by accurate and practicable electronic structure calculations. Therefore, it is feasible to conduct rational optimization for the transition metal catalysts by modulating the composition to modify electronic structure, further to regulate catalytic reaction rates that are usually depicted with turnover frequencies (TOFs).

In addition to the traditional industrial reactions, heterogeneous catalysis can also be extended to nanoscale, involving the catalytic reactions of nanocarbons. When a metal nanoparticle remarkably shrinks to contain only a limited quantity of atoms, its electron energy level would become discrete due to the quantum size effect.^[^ ^49^, ^50^ ^]^ Therefore, the efficient transfer of electrons and holes nonlocally to assist the nanometal catalysts may become the controlling step for the reactions. On the other hand, nanocarbons in sp^2^ hybridized state, like CNTs and graphene, are semimetals with a number of electrons available at the surface.^[^ ^22^ ^]^ They also possess extremely high mobility of carriers^[^ ^51^, ^52^ ^]^ and can transport electrons and holes equally at massless fermi velocity. All these properties enable nanocarbons to serve as heterogeneous catalysts themselves, meanwhile as electron and hole conductor for nanometals. Compared with transition metals, there is no d‐band in the electronic structure of carbon element, but sp^2^ nanocarbons own unique band structure. The local density of states (LDOS) and electronic structure at the edge should be considered first, but they are dynamically variable due to many‐body interactions, which is dependent on the topological defects, partial hydrogenation, oxygen termination, etching of metal nanoparticles, and other edge functionalization schemes.^[^ ^53^, ^54^, ^55^ ^]^ By contrast, the holistic bandgap of sp^2^ nanocarbon crystal is more uniform and tunable as a property of long‐range lattice. A broad range of bandgap exists in CNTs, which means that m‐CNTs possess zero or tiny bandgap, while s‐CNTs have obvious bandgap inversely proportional to diameter. Even for graphene, which is zero‐bandgap intrinsically,^[^ ^27^, ^28^ ^]^ bandgap can also be opened up by lateral confinement and is tunable.^[^ ^29^ ^]^ Consequently, the bandgap of sp^2^ nanocarbons is more important when investigating their own catalytic effect in the growth.

For the catalytic growth of nanocarbons, where nanocarbons are coupled with metal catalysts, they can serve as templates and catalysts themselves to play a role in modifying the electronic structure of metals as well as transporting carriers in a long‐range distance. All these endow the growth process with a complex characteristic, which means the growth of nanocarbons is a reaction partly catalyzed by one of its products, namely autocatalysis, which has been proposed in the growth of agglomerated and vertically aligned CNT,^[^ ^56^, ^57^ ^]^ fullerene,^[^ ^58^ ^]^ biosystems,^[^ ^59^, ^60^, ^61^ ^]^ and other fields.^[^ ^62^, ^63^, ^64^, ^65^, ^66^ ^]^ The autocatalysis characteristic in the growth of nanocarbons, i.e., the carbocatalysis growth, along with the tunable band structure, substantially provides a basis for the bandgap‐coupled template growth kinetics. If such kinetics can be verified, certain nanocarbons, whose bandgap can match well with the optimal growth kinetics, would grow superiorly with faster rate and perfect structure. Selective growth of specific kinds of CNTs and graphene can thus be achieved.

### 4.2 Bandgap‐Coupled Catalytic Growth Kinetics of Graphene

**Figure 5:**
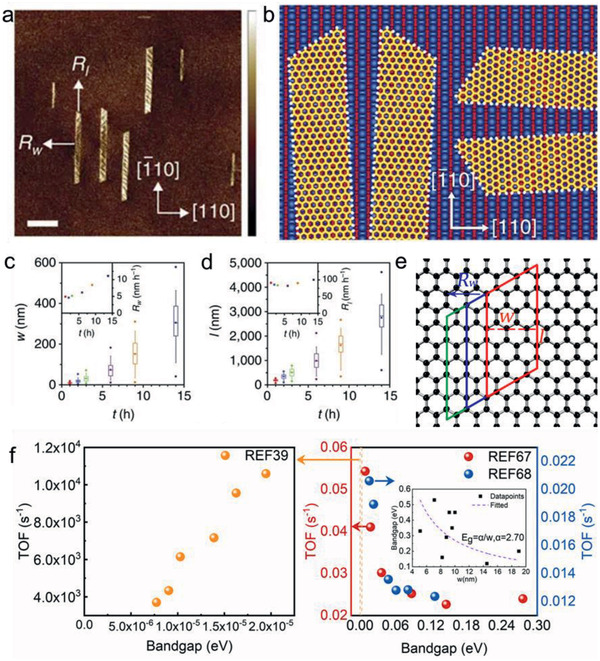
Bandgap‐coupled growth kinetics of GNRs and graphene. a) AFM image of GNRs on Ge(001), showing the shapes are parallelogram and isosceles trapezoid. Scale bar, 400 nm. b) Schematic illustration depicting the four orientations of as‐prepared nanoribbons, with armchair edges. c) Ribbon width *w*, d) length *l* plotted against growth time *t*. Horizontal lines in the boxes define the 25th, 50th, and 75th percentiles, whiskers indicate the 5th and 95th percentiles, circles define the range and squares give the mean. Insets are the mean rate *R_w_* and *R_l_*, respectively, plotted against *t*. a–d) Reproduced with permission.^[^ ^67^ ^]^ Copyright 2015, Springer Nature. e) Schematic illustration of the method to calculate TOF of *R_w_*. f) Plot of TOF against bandgap of graphene and GNRs in different CVD systems. Left: TOF versus equivalent bandgap of graphene.^[^ ^39^ ^]^ Right: TOF versus bandgap of GNRs.^[^ ^67^ ^,68]^

Although graphene is intrinsically zero‐bandgap, the bandgap would be opened up when the domain is narrowed down to a nanoribbon. It is important to precisely control the width (*w*) of GNRs so as to mediate their bandgap and electronics performances. The width‐related bandgap of GNRs could also impact the growth kinetics, which is exemplified by the anisotropic growth of GNRs on Ge(001) facets with controlled dynamics.^[^ ^67^ ^]^ The as‐prepared GNRs are narrow in width (even below 10 nm), with controllable morphology and precise armchair edges (**Figure** **5**a,b). The growth rate in the width direction *R_w_* is extremely slow, typically about 5 nm h^−1^, while that in length direction *R_l_* can be kept at about 90 nm h^−1^. As shown in Figure 5c,d, after several hours of growth, *R_w_* rises obviously after *w* exceeds about 30 nm and achieves above 10 nm h^−1^ at the time of 13.75 h, but *R_l_* maintains relatively constant. They attributed the increase in *R_w_* to the decreasing barrier of hydrocarbon attachment to the longer‐side ribbon edge, and *w* ≈ 30 nm was speculated to be the position where ribbons begin to outgrow the Ge terrace they nucleate on.

In order to reveal the dependence of catalytic growth kinetics on the bandgap, here we introduce the TOF, a classical concept in industrial catalysis, to depict the growth rate from a catalytic perspective and define it as the counts of carbon atoms adsorbing onto single active site per second. For the GNRs, the TOFs include two categories: one is the TOFs of carbon atoms adding onto length edge (corresponding to *R_w_*), and the other is the TOFs of carbon atoms adding onto width edge (corresponding to *R_l_*), between which the TOF of *R_w_* should be paid more attention to, as *R_w_* is the determinant rate for the bandgap variance. As Figure 5e manifests, there are three armchair sites in a distance range of 9*a*_C–C_ (*a*_C–C_ is the nearest‐neighbor carbon–carbon distance) at the length edge, if width increases a distance of $\sqrt{3}$ *a*_C–C_, there would be on average 13 carbon atoms adding onto the edge. As a result, TOF(*R_w_*) should be calculated by $13\;\times \frac{R_{w}}{\sqrt{3}a_{c-c}}\times \frac{1}{3}\;s^{-1}\;$. Based on the inversely proportional relationship, the dependence of bandgap on the width of GNRs can be simulated as *E*_g_ ≈ 2.70/*w*, obtained by fitting the data of widths and corresponding bandgaps of nine GNRs prepared in this growth system (Figure 5f inset).

As shown by the red data points in the right plane of Figure 5f, within the bandgap range from 8.85 × 10^−3^ eV (*w* = 305 nm) to 0.276 eV (*w* = 9.79 nm), TOF increases prominently as bandgap decreases, among which the maximum TOF is more than two times larger than the minimum one. It should be noted that the bandgap 8.85 × 10^−3^ eV is close to the boundary of semiconducting GNR and metallic graphene. Similarly, we can obtain the relationship between the TOF and bandgap in the armchair GNR growth from another work,^[^ ^68^ ^]^ also manifested in the right plane of Figure 5f. In a similar bandgap range, the TOF exhibits a similar trend, although the TOF values are incongruent due to different growth conditions, which indicates the effect of environment on the growth kinetics. For GNRs with ≈1 × 10^−3^ eV bandgap (*w* ≈ 2700 nm) or even larger size, they would generally behave just like a zero‐bandgap graphene in the growth kinetics.

For the graphene growth with higher rates, usually in the range of several micrometers to tens of micrometers per minute,^[^ ^39^, ^69^, ^70^ ^]^ the recorded graphene sizes generally begin with several tens of micrometers and continue to enlarge, but with a gradually decreased growth rate, which manifests a declining TOF trend as the equivalent bandgap decreases in the range of *E*_g_ < 10^−4^ eV, as shown in the left plane of Figure 5f. The above results need to be extrapolated further to the medium range, if a legible downward tendency of the TOF appears with the shrinking bandgap, it would be more evident that the growth kinetics of GNRs and graphene are strongly bandgap‐dependent.

### 4.3 Bandgap‐Coupled Autocatalytic Growth Kinetics of CNTs

Different from graphene, whose bandgap would vary as the size enlarges, the bandgap of a CNT generally maintains invariable after the formation of infant tube marked by a mature cap with six pentagons, as long as the atomic assembly templated by the tube edge is in the correct way. This feature makes it more definite to investigate the bandgap‐coupled growth kinetics of CNTs. In fact, numerous investigations have reported narrow diameter distributions of CNTs prepared by CVD. Recently, the achievement of colorful SWNT film by Kauppinen et al.^[^ ^71^ ^]^ has drawn much attention to the enrichment of specific kinds of CNTs by modulating the growth environment. Furthermore, if superior TOF and durable survival time exist in the partial CNT species with specific bandgap, i.e., growing faster in a longer lifetime, such a strategy could be utilized to achieve high‐purity selective growth of specific CNTs, just like the evolution in biosystem, which may also happen in the template growth of sp^2^ nanocarbons.

#### 4.3.1 Cloning Growth of Chirality‐Specific CNTs with Carbocatalysis

**Figure 6:**
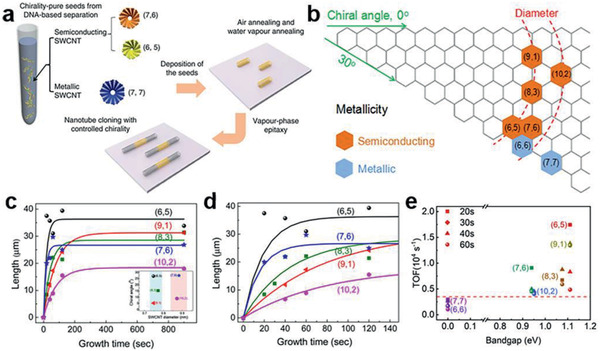
CNT cloning growth and the bandgap‐coupled kinetics. a) Schematic illustration of the cloning growth for chirality‐determined CNTs. a) Reproduced with permission.^[^ ^73^ ^]^ Copyright 2012, Springer Nature. b) Chirality map of the seven kinds of single‐chirality CNTs used for cloning growth. c) Time evolution of length and fitted curves for five chiral s‐CNTs with growth times of 20, 30, 40, 60 s, 2, and 15 min. Inset, chiral angle versus diameter of the above 5 s‐CNTs, showing that they belong to two subgroups with similar diameters. d) Enlarged plot of the initial growth period in (c). b–d) Reproduced with permission.^[^ ^74^ ^]^ Copyright 2012, American Chemical Society. e) Plot of TOF versus bandgap for seven single‐chirality CNTs, where different shapes represent data at different times and different colors represent data of seven different single‐chirality CNTs.

Cloning growth of CNTs, which is a rational approach to produce CNTs with predefined chirality by using open‐end CNTs as “seeds/catalysts” via epitaxial growth,^[^ ^72^, ^73^, ^74^ ^]^ directly demonstrates the autocatalysis performance of a CNT seed without metal catalysts. The CNT seeds with a definite chirality can be directly obtained by chemical or biological separations (**Figure** **6**a), thus the nucleation stage can be circumvented, making it feasible to focus on growth kinetics of different single‐chirality CNTs.

Zhou et al.^[^ ^74^ ^]^ studied cloning growth of seven kinds of chirality‐specific CNTs with small diameters from 0.76 to 0.96 nm, including metallic and semiconducting species (Figure 6b), and measured their epitaxial growth rates. Specially, they discovered that the two metallic CNTs are generally with obviously shorter lifetime and length, while the semiconducting ones favor longer growth duration. As demonstrated in Figure 6c,d, it is evident that s‐CNTs possess distinct growth kinetics during the initial growth period.

To investigate the relationship between the bandgap of CNT and the growth kinetics, we similarly depict the growth rate by TOF, which represents the counts of C2 assembled onto one circumferential active site per second. And the method to derive TOF for a CNT with specific chirality from length rate is TOF = *R* × *Q*_atom_/*m*,^[^ ^75^ ^]^ where *R* is length growth rate, *Q*_atom_ is the number of carbon atoms within a 1 nm long CNT wall and *m* is the chiral index. It is necessary to note that elongation can happen on both ends of the CNT seed during the cloning growth, different from the metal‐catalyzed CVD. Consequently, to obtain the TOF of cloning growth, the calculated growth rates derived from CNT length should be divided by two, and the analysis results are summarized in Figure 6e, where the TOFs of chiral s‐CNTs can be identified to be evidently higher than those of armchair m‐CNTs. The bandgap‐coupled growth kinetics of CNT cloning growth is demonstrated clearly by the different results between the m‐CNTs and the s‐CNTs.

The cloning growth results confirmed the template autocatalysis or carbocatalysis ability in CNT growth. Besides, nanometer‐size graphene crystals^[^ ^76^, ^77^ ^]^ have also been utilized as seeds in CVD reproduction of graphene.

#### 4.3.2 Environment‐Related Narrow Bandgap Distribution of CNTs

In fact, template autocatalysis of nanocarbons is sensitive to the environment, which can be manifested by the example of one source of the CNT discovery.^[^ ^78^, ^79^ ^]^ In the hydrocarbon steam cracking process, CNTs and carbon filaments were found and deduced to originate from the CVD process on transition metal, where the existence of Fe can readily promote the CNT growth by 1000 times in proper conditions. On the contrary, even ppm‐level amount of S compound additives, such as CS_2_, would lower the reaction rate by orders of magnitude, which is a practical method in petrochemical industry for preventing coking.^[^ ^80^ ^]^ Obviously, environment conditions would profoundly impact the reaction rate of nanocarbons autocatalysis, further to influence other aspects.

It should be noted that the relative abundance *A* of CNTs with certain chirality or bandgap can be described as *A* ∼ *N* × *R*, where *N* is nucleation probability and *R* is growth rate. For the growth systems with liquid catalysts, the catalysts are fully compliant and conform to the profile of the CNT edge, thus the growth is dominated by the elongation process and kinetics. Consequently, the bandgap distribution of final as‐prepared CNTs can be a reliable parameter to mediately verify the relationship between the atomic assembly rate and bandgap.

**Figure 7:**
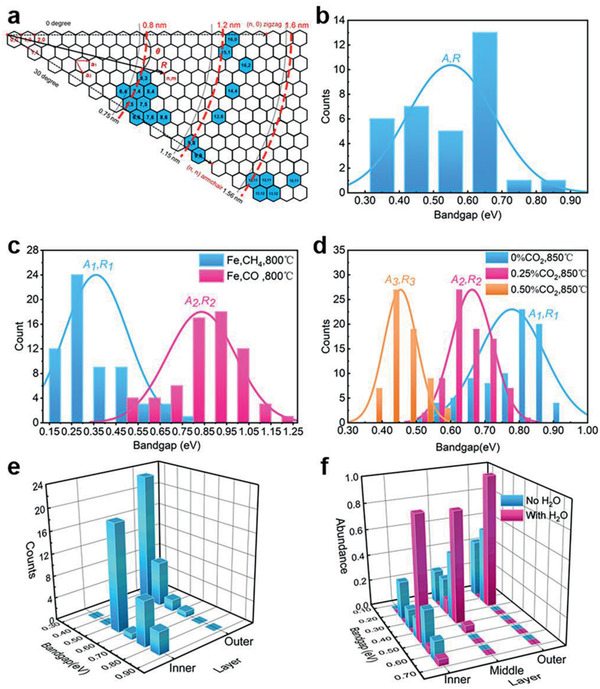
Environment‐related bandgap enrichment of CNTs. a) (*n*, *m*) SWNT species (blue hexagons) achieved in chirality selection synthesis, which are clustered around three diameters of 0.8, 1.2, and 1.6 nm, labeled by red dashed line. Adapted with permission.^[^ ^81^ ^]^ Copyright 2013, Elsevier. b–f) Narrow bandgap distribution of CNTs in different liquid‐catalyst systems showing different preference of abundance *A* and growth rate *R* on bandgap. All data are transformed from chiral indices obtained by ED. b) CNTs prepared with Fe catalyst, CO carbon source at 800 °C.^[^ ^82^ ^]^ c) CNTs prepared with Fe catalyst, at 800 °C. Different carbon sources result in the difference of bandgap distribution.^[^ ^83^ ^]^ d) CNTs prepared by floating catalyst CVD with ferrocene catalyst precursor, CO carbon source at 850 °C. A tiny amount of CO_2_ and different CO_2_ concentrations result in the difference of bandgap distribution.^[^ ^71^ ^]^ e) Each layer of DWNTs prepared by our group with Fe catalyst, at 1005 °C.^[^ ^84^, ^85^ ^]^ f) Each layer of TWNTs prepared by our group with Fe catalyst, at 1005 °C. A small amount of H_2_O result in the shift of bandgap distribution.^[^ ^84^, ^85^ ^]^

In a previous review, Chen et al.^[^ ^81^ ^]^ found that the CNTs species having been enriched were clustered approximately around three separate diameters at 0.8, 1.2, and 1.6 nm (**Figure** **7**a). As most of these enriched CNTs are semiconducting species, this phenomenon may be related to their specific bandgaps considering the inverse relation between bandgap and diameter, especially for enriched (14,4), (16,2) CNTs, which cannot be explained felicitously by existing mechanisms. Furthermore, it is meaningful to analyze the explicit chirality distribution determined by electron diffraction (ED) of CNTs prepared in liquid‐catalyst CVD systems under various conditions,^[^ ^71^, ^82^, ^83^, ^84^, ^85^ ^]^ including the millimeter‐ and centimeter‐long double‐walled nanotubes (DWNTs) and triple‐walled nanotubes (TWNTs) achieved in our Fe‐catalyzed CVD experiments. The chirality distribution can be utilized to deduce corresponding bandgap distribution, whose results are summarized in Figure 7b–f. It is clear that the bandgap distributions are concentrated in specific narrow ranges despite of varied growth systems, which is the same for each layer of DWNTs and TWNTs (Figure 7e,f). Such preferences of abundance *A* corroborate that the growth rates *R* would stand out merely for the CNTs within specific bandgap range, addressing a dependence of growth rate on the electronic structure. As manifested in Figure 7b–f, the bandgap distribution would react sensitively to any change of the environment parameters in such systems. For instance, even a tiny amount of CO_2_ and its concentration difference in the gas atmosphere would induce prominent shift of the growth rate preference and the final bandgap enrichment, creating colorful CNT films.^[^ ^71^ ^]^ Our investigations also revealed that introducing little amount (≈0.5%) of H_2_O can also conspicuously narrow down and alter the bandgap distribution of each tube layer in TWNTs.^[^ ^84^, ^85^ ^]^ All these actually reveal that the chirality and bandgap of CNTs would evolve into specific dominant populations under the impact of varied environments.

Another noteworthy phenomenon in our liquid‐Fe‐catalyst CVD is that the chiral index distributions of all layers in DWNTs and TWNTs manifest a striking enrichment on near‐armchair and near‐(2*m*, *m*) species, which are thought to own fastest growth rate in the two edge‐related CNT growth kinetics models expounded above, respectively. It seems irrational because the two kinetics models are built on different catalyst states, and requires more thorough exploration.

#### 4.3.3 Interlocking between the Growth Kinetics and the Bandgap of CNTs

Recently, our investigation has revealed a clear interlocking relationship between growth kinetics of ultralong CNTs and their bandgap,^[^ ^75^ ^]^ where the growth follows vapor–liquid–solid mode. Hence, the kinetics and the template atomic assembly dominate the growth process. We have used isotope switching method and micro‐Raman characterization to determine the growth rates and TOFs of individual CNTs.

**Figure 8:**
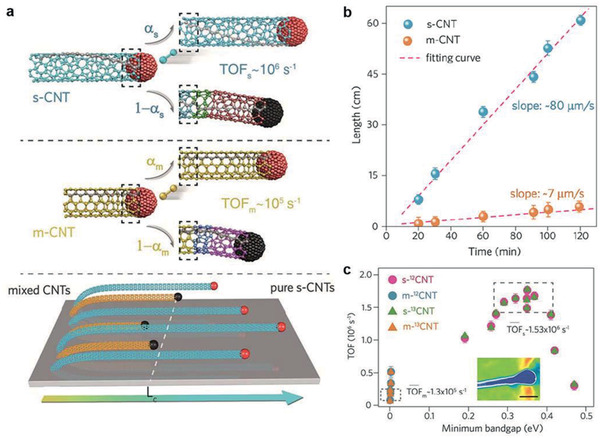
Atomic‐assembly growth kinetics of CNTs and its interlocking with the bandgap. a) Schematic illustration of the spontaneous purification of s‐CNTs by TOF differences between m‐ and s‐CNTs. b) Length plotted against growth time of m‐ and s‐CNTs, showing different growth rates. Error bars represent standard deviation of ten longest m‐ or s‐CNTs at different growth times. c) Minimum bandgap of concentric layers for few‐walled CNTs versus corresponding TOF. Inset is an AFM image on the tip of a long CNT, scale bar, 10 nm. a–c) Reproduced with permission.^[^ ^75^ ^]^ Copyright 2019, Springer Nature.

All CNTs increase in length linearly with growth time and obey the Schulz–Flory distribution,^[^ ^20^ ^]^ which means that the probability of each carbon dimer catalytically integrating onto the CNTs all equals in the growth and the CNT quantity would decrease exponentially with the length. Half decay length, defined as the length where the CNT quantity decreases by half compared to that near the catalyst, is used to evaluate the CNT decay behavior. The experimental results revealed that CNTs could be categorized into two types due to their striking difference in TOF. One type of CNTs grow at a faster constant rate with average TOF of ≈1.5 × 10^6^ s^−1^, and thus with longer length. On the contrary, the other type of CNTs possess slower constant growth rate and decay more easily, whose average TOF (≈1.3 × 10^5^ s^−1^) and half decay length are all one order of magnitude lower than the former type (**Figure** **8**a,b). Raman and electronic measurements have manifested that the first type of CNTs are semiconducting and the second type are metallic or with at least one metallic layer. Furthermore, the s‐CNTs within specific bandgap range exhibit higher TOF than other s‐CNTs (Figure 8c). The volcano‐type dependence of TOF on the bandgap has been demonstrated, just as the Brønsted–Evans–Polanyi relationship in the transition metal catalysis.^[^ ^43^, ^44^, ^45^ ^]^ By utilizing such spontaneous rate‐selected purification for semiconducting CNTs, it is believed that 99.9999% s‐CNTs purity can be accomplished at the length of 154 mm. By optimizing the operation conditions, more than ten billion steps of dimer addition without deactivation could be accomplished. Therefore, we have obtained highly pure s‐CNTs with the longest length of 650 mm, providing a reliable approach to synthesize high‐purity s‐CNTs with a narrow bandgap distribution.

More importantly, our work has highlighted the interlocking relationship between electronic structure and catalytic growth kinetics, validating the bandgap‐coupled catalytic growth of CNTs robustly, with an explicit volcano dependence. Under certain growth environment, once the CNT growth passes the infant stage, the interlocking relationship between the bandgap and growth rate would be built, making the ultrafast growth of bandgap‐specific CNTs highly efficient, as the process has witnessed more than ten billion independent template assembly steps of carbon dimers, with sustained long‐range catalytic action and carrier transport of nanocarbons.

## 5 Defect‐Induced Disturbance for the Bandgap‐Coupled Catalytic Growth

### 5.1 The Protection for Perfect sp^2^ Structure against Topological Defects

**Figure 9:**
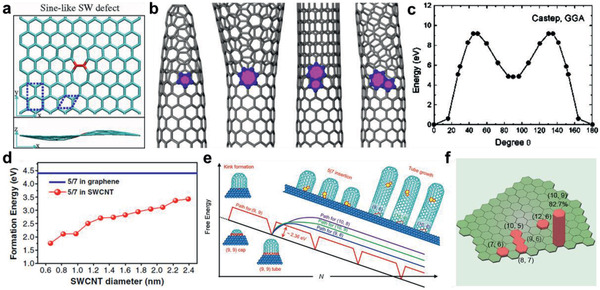
The formation of topological defects. a) Top and side views of the sine‐like Stones–Wales defect causing local curvature in graphene sheet. a) Adapted with permission.^[^ ^86^ ^]^ Copyright 2009, American Physical Society. b) An isolated pentagon turns a (10,0) SWNT into a cone and a heptagon turns it into a horn. And different orientations of 5|7 pair cause different intramolecular junctions. c) The formation energy of Stones–Wales defect by rotating one bond by 90°. c) Reproduced with permission.^[^ ^90^ ^]^ Copyright 2005, American Physical Society. d) The formation energies of a 5|7 pair in graphene and in SWNTs versus the diameter. b,d) Reproduced with permission.^[^ ^89^ ^]^ Copyright 2012, American Physical Society. e) Schematic illustration about the energy barriers of (9,9) tube growth and different chirality transformations with different 5|7 pair orientations. f) Chirality distribution of mutation growth influenced by the stability of different 5|7 pair orientations. Reproduced with permission.^[^ ^96^ ^]^ Copyright 2019, Elsevier.

Perfect sp^2^ nanocarbons own a zero Gaussian curvature and ordered electronic structure intrinsically, while the appearance of topological defects, such as pentagon, heptagon, and other nonhexagonal rings, can locally change Gaussian curvature into positive or negative and alter the structure. Local curvature in planar graphene sheet could be induced by topological defects (**Figure** **9**a),^[^ ^86^, ^87^ ^]^ among which the most typical one is the so‐called Stone–Wales (SW) defect,^[^ ^88^ ^]^ formed by rotating one bond and transforming four hexagons into two pentagons and two heptagons(55‐77), while other defects include single vacancy (5‐9), divacancy (5‐8‐5), and so on. For CNTs, pentagon and heptagon defects can only exist in the form of pentagon‐heptagon pair (5|7) to maintain a tubular morphology according to Euler's rule. In fact, an isolated pentagon would turn a SWNT into a sharp cone and an isolated heptagon turns it into a horn (Figure 9b).^[^ ^89^ ^]^ Thus, the 5|7 pair is the simplest topological defect in CNT, which can change the edge structure and the chirality of a CNT, forming (*n*, *m*) − (*n* + 1, *m* − 1), (*n*, *m*) − (*n* + 1, *m*) or (*n*, *m*) − (*n*, *m* + 1) intramolecular junctions, according to different orientations of 5|7 pair.

The formation of topological defects is usually accompanied with high energy barriers, which builds topological protection for perfect sp^2^ structure. For graphene, the SW defect has a fairly large formation energy *E*_f_ ≈ 5 eV (Figure 9c),^[^ ^86^, ^90^ ^]^ while the *E*_f_ of 5|7 pair can be as high as 4.4 eV.^[^ ^89^ ^]^ Nevertheless, curvature would lower the defect formation energy. Due to the conforming of graphene to the substrate, any curvature or roughness on the substrate would decrease the defect formation energy and lead to defects in the sp^2^ network of graphene.^[^ ^89^, ^91^, ^92^, ^93^ ^]^ For CNTs, Ding and co‐workers^[^ ^89^ ^]^ have investigated the 5|7 pair, the most energetically preferred topological defect, and found that the formation energy would drop remarkably as the diameter decreases, with a 3.4 eV value for *d* = 2.3 nm and 2.1 eV for *d* = 1.0 nm (Figure 9d). Thus, the 5|7 defect would more likely appear in small‐diameter CNTs, accounting for the extensive experiment results that small‐diameter CNTs can hardly achieve ultralong length. Besides, the formation energy of topological defect would also be altered by its orientation. A 5|7 pair in CNT would cause different intramolecular junctions according to its different orientations, which would meanwhile make a difference to the stability of junctions and further chirality distribution. Calculations have elucidated that the SWNT junctions where 5|7 pairs are aligned along the nanotube axis would be more stable than those with 5|7 pairs distributed along the cylindrical circumference.^[^ ^94^, ^95^ ^]^ It may result from the sixfold symmetry of CNT hexagonal network. An recent investigation about chirality transformation, which is implemented by introducing 5|7 pair into infant (*n*, *n*) tubes, obtained dominant (*n*, *n* − 1) enrichment rather than (*n* + 1, *n* − 1),^[^ ^96^ ^]^ the former of which corresponds to a 5|7 pair aligned along the nanotube axis, certifying the calculation that the 5|7 pairs along tube axis are more likely to occur (Figure 9e,f).

Furthermore, the calculation results showed that different metal catalysts could assist with defect healing in CNT growth, among which Fe is the most helpful.^[^ ^89^ ^]^ With efficient defect healing, another protection for perfect structure, the low limit of 5|7 defect concentration can reach 10^−8^ to 10^−11^ in an optimal condition, corresponding to a defect‐free SWNT with 0.1–100 cm length. It should also be noted that the results indicated a faster growth rate would obstruct the defect healing.

### 5.2 Impact of Topological Defects on the Structures and Catalytic Growth

**Figure 10:**
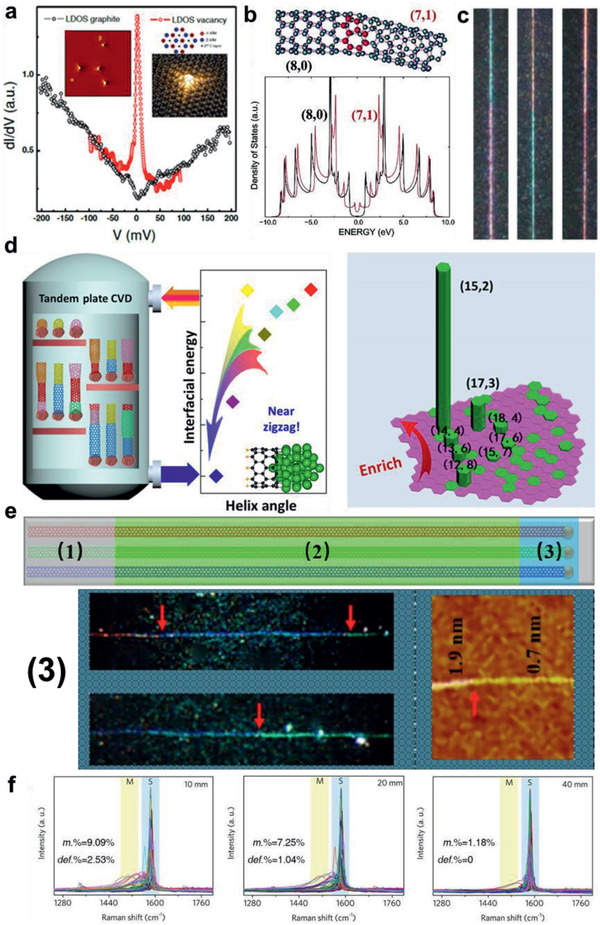
Defect‐induced interference for the electronic structures and the bandgap‐coupled growth. a) Tunneling conductance spectra acquired on the single vacancy of graphene compared with pristine graphene. Inset: Left is STM topography of the ion‐irradiated graphene. Right are schematic of the graphene and 3D view of a single vacancy. Adapted with permission.^[^ ^97^ ^]^ Copyright 2010, American Physical Society. b) Atomic structure of an (8, 0)‐(7, 1) CNT intramolecular junction and the corresponding electron density of states. Reproduced with permission.^[^ ^102^ ^]^ Copyright 2002, American Chemical Society. c) Typical Rayleigh true‐color images of CNTs with intramolecular junctions. Adapted with permission.^[^ ^105^ ^]^ Copyright 2015, Springer Nature. d) Schematic illustration of the chirality‐transformation preparation of CNTs using temperature perturbations, and the final chirality distribution analyzed by Raman spectroscopy. A dominating s‐CNT distribution is shown. Adapted with permission.^[^ ^106^ ^]^ Copyright 2016, American Association for the Advancement of Science. e) Schematic illustration of CNTs grown with tip mode and characterization images acquired on the end part of CNTs. Several chirality changes are shown in the Rayleigh images with diameter change shown in the AFM image. f) The defective CNT percentage (def.%) and m‐CNT percentage (m%) at specific length in the kinetic growth, measured by Raman spectra. Adapted with permission.^[^ ^75^ ^]^ Copyright 2019, Springer Nature.

Once topological defects appear in sp^2^ nanocarbons, it would impact the electronic structure. Single vacancy,^[^ ^97^ ^]^ divacancy,^[^ ^98^ ^]^ and SW defect^[^ ^99^ ^]^ in graphene would modify the local electronic structure drastically, which can be unveiled by theoretical calculation and LDOS map of scanning tunneling microscope (STM) measurement (**Figure** **10**a). Furthermore, it has been demonstrated that holistic bandgap would be also altered, even bandgap closing, when the defect scattering is above a threshold.^[^ ^100^, ^101^ ^]^ In fact, the 5|7‐defect‐induced bandgap change in CNTs could be embodied as the chirality change and intramolecular junctions (Figure 10b),^[^ ^102^, ^103^, ^104^, ^105^ ^]^ even a transformation from s‐CNT to m‐CNT or the opposite, shown as the color change in Rayleigh characterization (Figure 10c).

Defect‐induced change in band structure would certainly influence the growth kinetics of sp^2^ nanocarbons, considering the interlocking between the growth kinetics and bandgap. It would break the efficient interlocking and bring about unstable growth rate. Thus, the defective graphene and CNTs cannot develop into those with large size or ultralong length. Taking CNT as an example, if a 5|7 pair defect arose during the growth of a CNT and caused a chirality change, especially for the s‐ to m‐CNT transformation, which means substantial change of bandgap and TOF by orders of magnitude, the CNT would yield a drastically reduced growth rate, or stop growth. Consequently, when Zhang and co‐workers investigated the chirality enrichment of CNTs after multiple chirality changes induced by temperature perturbations,^[^ ^106^ ^]^ the results demonstrated an absolute s‐CNT enrichment (Figure 10d). The dominant s‐CNT distribution in aforementioned chirality‐transformation growth investigation^[^ ^96^ ^]^ has also confirmed that the s‐ to m‐CNT transformation is not as favored as the opposite one. Moreover, the defect‐induced unstable growth can also be exemplified by our experimental observations. As shown in Figure 10e, Rayleigh images of individual CNTs obeying tip growth mode manifest that the colors of CNTs changed once or several times at the end, indicating that the growing CNTs would approach death after several chirality changes induced by topological defects. Our statistical results of kinetic growth of ultralong CNTs^[^ ^75^ ^]^ evidently show that the percentage of defective CNTs would decrease sharply as the length increases (Figure 10f), arising from the defect‐induced disturbance for growth and resultant inferiority in kinetic competence than perfect CNTs.

To summarize, topological defects would induce disturbance for the bandgap‐coupled catalytic growth and break the interlocking between bandgap and atomic assembly rate. It also demonstrates that the interlocking relation can provide directions for selective synthesis of atomically perfect CNTs and graphene.

## 6 Summary and Prospects

To achieve large‐scale superior applications of CNTs and graphene, catalytic CVD growth is the most feasible synthesis approach for high‐purity and defect‐free sp^2^ nanocarbons. The fairly high formation energies of topological defects, typically 4.4 eV for the formation of a 5|7 defect in graphene, build a topological protection for the sp^2^ structure basically, which makes fundamental significance for the controlled growth of sp^2^ nanocarbons with ultralarge scale and perfect structure. The protection would be impacted profoundly by the curvature of the sp^2^ surface. About 1.3 nm change in radial curvature of substrate or CNT diameter would cause a prominent decrease of defect formation energy and an increase of defect density by about 10^6^ times, which make it more difficult to obtain small‐diameter CNTs with ultralong length and perfect structure.

On the other hand, unique properties endow nanocarbons with the ability to serve as template catalysts with tunable bandgap and play a role in the catalytic growth. Existing catalytic theories of nanocarbons hold that local sites are important, especially the current growth kinetics, which all focus on the kinks and edges, while the effect of autocatalysis and holistic electronic structure is ignored. In this article, we consider the catalytic kinetics from the perspective of template autocatalysis and holistic electronic structure. Different representative examples involving various types of catalytic growth of CNTs and graphene are reviewed, to verify the bandgap‐dependent template autocatalysis growth kinetics. Finally, we review our recent work, which have unveiled an interlocking between the atomic assembly rate and bandgap with a volcano dependence in the growth of ultralong CNTs, in which autocatalysis promotes faster growth and high‐purity synthesis of certain CNTs. This work shows a long‐range catalytic action of CNTs and provides a novel perspective to understand catalytic growth mechanisms of nanocarbons.

We have revealed the bandgap‐coupled kinetics in the catalytic growth of sp^2^ nanocarbons, bridging the carbocatalysis, and holistic electronic structure. Nevertheless, as the cloning growth revealed, the small diameters of seeded CNTs and nonmetal‐catalytic characteristics induce lower defect formation energy, higher activation energy for growth, and absence of efficient defect healing by metal catalyst, resulting in relative easier defect generation, lower growth rate, shorter survival time, and resultant shorter perfect‐structure length, when compared with the metal‐catalyzed CVD growth. It demonstrates that it is difficult to merely use CNT itself to catalyze high‐efficiency and high‐quality growth. Nevertheless, when metal catalysts and sp^2^ nanocarbons are combined as a whole, nanocarbons and their bandgap also play a role in the complicated template autocatalysis. As long as the interlocking between bandgap and kinetics is maintained well, the catalytic growth would be highly stable and efficient, keeping even tens of billions of hexagonal rings consistent. Since specific bandgap range prevails in the bandgap‐coupled growth kinetics, various kinds of CNTs could be prepared selectively by adjusting growth conditions, which can modulate the optimal bandgap. On the other hand, the interlocking would be broken by the local topological defects in nanocarbons, which can observably alter the holistic band structure, and disturb the catalytic growth dramatically.

Based on the demonstrations all above, we think that the following issues should be focused on further. First, tunable bandgap and template autocatalysis ability of sp^2^ nanocarbons that were ignored previously, have been verified to play a role in the kinetic growth. Carbocatalysis and volcano dependence of the atomic assembly rate on the bandgap bring about structural selectivity. Such couplings of bandgap and growth, structure and template autocatalysis would be of vital significance for structure‐controlled preparation in the future.

Another noteworthy thing is the impact of growth environment for the CNTs surviving and dominating in it. Due to the environmental selection, the CNTs with sustained catalytic activity evolve into dominated populations even exhibit specific colors, like what happens in biosystem evolution. Thus, the influence of the growth environment should be paid more attention from a different perspective.

Then some issues about defect‐induced break of the interlocking are still unclear. According to the calculations about CNT,^[^ ^89^ ^]^ a faster growth rate would obstruct the defect healing. In fact, we have prepared 65 cm long perfect CNTs at an ultrafast growth rate of ≈80 µm s^−1^. The calculation indicated that the 5|7 defect concentration under our growth conditions should be about 10^−8^, markedly deviating from our actual results that showed no larger than 10^−12^. Considering that most of our as‐prepared CNTs are DWNTs and TWNTs, the formation energy of 5|7 defect in outer walls with larger diameters may be large enough to restrain defect formation. But the ultralow defect concentration in small‐diameter inner walls seems to be unreasonable, which perhaps can be interpreted as a protection from outer walls. In addition, we have observed that the chiral indices of three walls in a TWNT maintained respective consistence in a 35 mm range and all changed almost at the same location,^[^ ^84^ ^]^ which showed the synchronism of chirality change among the three walls to prevent their mutual penetration. Based on the above phenomenon, the deep mechanisms behind the defect formation, the interaction of different tube walls and their impact on the interlocking relationship are still indistinct and need to be further investigated.

Notwithstanding graphene and CNTs have long been regarded as ideal materials for various promising applications there are still many obstacles, especially the indistinction of fundamental mechanisms dominating the high‐quality preparation. With relentless efforts in nanocarbon research, we expect that the catalytic growth mechanisms and the defect formation of nanocarbons would be more explicit, controlled synthesis of perfect CNTs and graphene would be more reliable to fulfill the anticipated potential and develop more novel applications.

## Conflict of Interest

The authors declare no conflict of interest.

## References

[1] S. Iijima , Nature 1991, 354, 56.

[2] K. S. Novoselov , A. K. Geim , S. V. Morozov , D. Jiang , Y. Zhang , S. V. Dubonos , I. V. Grigorieva , A. A. Firsov , Science 2004, 306, 666. 15499015 10.1126/science.1102896

[3] A. K. Geim , K. S. Novoselov , Nat. Mater. 2007, 6, 183. 17330084 10.1038/nmat1849

[4] A. K. Geim , Science 2009, 324, 1530. 19541989 10.1126/science.1158877

[5] F. Bonaccorso , Z. Sun , T. Hasan , A. C. Ferrari , Nat. Photonics 2010, 4, 611.

[6] A. D. Franklin , Nature 2013, 498, 443. 23803839 10.1038/498443a

[7] M. S. Dresselhaus , G. Dresselhaus , A. Jorio , Annu. Rev. Mater. Res. 2004, 34, 247.

[8] T. Durkop , S. A. Getty , E. Cobas , M. S. Fuhrer , Nano Lett. 2004, 4, 35.

[9] R. H. Baughman , A. A. Zakhidov , W. A. de Heer , Science 2002, 297, 787. 12161643 10.1126/science.1060928

[10] H. Dai , Cheminform 2003, 34, 1035.

[11] Y. Bai , R. Zhang , X. Ye , Z. Zhu , H. Xie , B. Shen , D. Cai , B. Liu , C. Zhang , Z. Jia , S. Zhang , X. Li , F. Wei , Nat. Nanotechnol. 2018, 13, 589. 29760522 10.1038/s41565-018-0141-z

[12] R. Zhang , Z. Ning , Y. Zhang , Q. Zheng , Q. Chen , H. Xie , Q. Zhang , W. Qian , F. Wei , Nat. Nanotechnol. 2013, 8, 912. 24185944 10.1038/nnano.2013.217

[13] Y. Cao , V. Fatemi , S. Fang , K. Watanabe , T. Taniguchi , E. Kaxiras , P. Jarillo‐Herrero , Nature 2018, 556, 43. 29512651 10.1038/nature26160

[14] C. Qiu , Z. Zhang , M. Xiao , Y. Yang , D. Zhong , L. Peng , Science 2017, 355, 271. 28104886 10.1126/science.aaj1628

[15] M. M. Shulaker , G. Hills , R. S. Park , R. T. Howe , K. Saraswat , H. S. P. Wong , S. Mitra , Nature 2017, 547, 74. 28682331 10.1038/nature22994

[16] G. Hills , C. Lau , A. Wright , S. Fuller , M. D. Bishop , T. Srimani , P. Kanhaiya , R. Ho , A. Amer , Y. Stein , D. Murphy , Arvind , A. Chandrakasan , M. M. Shulaker , Nature 2019, 572, 595. 31462796 10.1038/s41586-019-1493-8

[17] M. M. Shulaker , G. Hills , N. Patil , H. Wei , H. Y. Chen , H. S. P. Wong , S. Mitra , Nature 2013, 501, 526. 24067711 10.1038/nature12502

[18] F. Yang , X. Wang , D. Zhang , J. Yang , D. Luo , Z. Xu , J. Wei , J. Q. Wang , Z. Xu , F. Peng , X. Li , R. Li , Y. Li , M. Li , X. Bai , F. Ding , Y. Li , Nature 2014, 510, 522. 24965654 10.1038/nature13434

[19] S. Zhang , L. Kang , X. Wang , L. Tong , L. Yang , Z. Wang , K. Qi , S. Deng , Q. Li , X. Bai , F. Ding , J. Zhang , Nature 2017, 543, 234. 28199307 10.1038/nature21051

[20] R. Zhang , Y. Zhang , Q. Zhang , H. Xie , W. Qian , F. Wei , ACS Nano 2013, 7, 6156. 23806050 10.1021/nn401995z

[21] D. Su , S. Perathoner , G. Centi , Chem. Rev. 2013, 113, 5782. 23721498 10.1021/cr300367d

[22] D. Su , J. Zhang , B. Frank , A. Thomas , X. Wang , J. Paraknowitsch , R. Schlogl , ChemSusChem 2010, 3, 169. 20127789 10.1002/cssc.200900180

[23] X. Duan , H. Sun , S. Wang , Acc. Chem. Res. 2018, 51, 678. 29494126 10.1021/acs.accounts.7b00535

[24] J. Zhang , X. Liu , R. Blume , A. Zhang , R. Schloegl , D. Su , Science 2008, 322, 73. 18832641 10.1126/science.1161916

[25] H. Yang , S. Song , R. Rao , X. Wang , Q. Yu , A. Zhang , J. Mol. Catal. A: Chem. 2010, 323, 33.

[26] J. Tan , J. Cui , X. Cui , T. Deng , X. Li , Y. Zhu , Y. Li , ACS Catal. 2015, 5, 7379.

[27] A. H. Castro Neto , F. Guinea , N. M. R. Peres , K. S. Novoselov , A. K. Geim , Rev. Mod. Phys. 2009, 81, 109.

[28] F. Schwierz , Nat. Nanotechnol. 2010, 5, 487. 20512128 10.1038/nnano.2010.89

[29] Y. Li , P. Cheol‐Hwan , S. Young‐Woo , M. L. Cohen , S. G. Louie , Phys. Rev. Lett. 2007, 99, 186801. 17995426 10.1103/PhysRevLett.99.186801

[30] T. Kato , R. Hatakeyama , Nat. Nanotechnol. 2012, 7, 651. 22961304 10.1038/nnano.2012.145

[31] V. Barone , O. Hod , G. E. Scuseria , Nano Lett. 2006, 6, 2748. 17163699 10.1021/nl0617033

[32] Y. W. Son , M. L. Cohen , S. G. Louie , Phys. Rev. Lett. 2006, 97, 216803. 17155765 10.1103/PhysRevLett.97.216803

[33] X. Li , X. Wang , L. Zhang , S. Lee , H. Dai , Science 2008, 319, 1229. 18218865 10.1126/science.1150878

[34] D. Querlioz , Y. Apertet , A. Valentin , K. Huet , A. Bournel , S. Galdin‐Retailleau , P. Dollfus , Appl. Phys. Lett. 2008, 92, 042108.

[35] M. Ouyang , J. L. Huang , C. L. Cheung , C. M. Lieber , Science 2001, 292, 702. 11326093 10.1126/science.1058853

[36] V. V. Deshpande , B. Chandra , R. Caldwell , D. S. Novikov , J. Hone , M. Bockrath , Science 2009, 323, 106. 19119228 10.1126/science.1165799

[37] R. Saito , G. Dresselhaus , M. S. Dresselhaus , Phys. Rev. B 2000, 61, 2981.

[38] Q. Yuan , H. Hu , F. Ding , Phys. Rev. Lett. 2011, 107, 155425. 10.1103/PhysRevLett.107.156101 22107305

[39] T. Ma , W. Ren , X. Zhang , Z. Liu , Y. Gao , L. Yin , X. Ma , F. Ding , H. Cheng , Proc. Natl. Acad. Sci. USA 2013, 110, 20386. 24297886 10.1073/pnas.1312802110 PMC3870701

[40] F. Ding , A. R. Harutyunyan , B. I. Yakobson , Proc. Natl. Acad. Sci. USA 2009, 106, 2506. 19202071 10.1073/pnas.0811946106 PMC2637275

[41] R. Rao , D. Liptak , T. Cherukuri , B. I. Yakobson , B. Maruyama , Nat. Mater. 2012, 11, 213. 22286334 10.1038/nmat3231

[42] V. I. Artyukhov , E. S. Penev , B. I. Yakobson , Nat. Commun. 2014, 5, 4892. 25224858 10.1038/ncomms5892

[43] F. Abild‐Pedersen , J. Greeley , F. Studt , J. Rossmeisl , T. R. Munter , P. G. Moses , E. Skúlason , T. Bligaard , J. K. Nørskov , Phys. Rev. Lett. 2007, 99, 16105. 10.1103/PhysRevLett.99.016105 17678168

[44] A. Logadottir , T. H. Rod , J. K. Nørskov , B. Hammer , S. Dahl , C. J. H. Jacobsen , J. Catal. 2001, 197, 229.

[45] T. Bligaard , J. K. Nørskov , S. Dahl , J. Matthiesen , C. H. Christensen , J. Sehested , J. Catal. 2004, 224, 206.

[46] J. K. Nørskov , Prog. Surf. Sci. 1991, 38, 103.

[47] A. Nilsson , L. G. M. Pettersson , B. Hammer , T. Bligaard , C. H. Christensen , J. K. Nørskov , Catal. Lett. 2005, 100, 111.

[48] B. Hammer , J. K. Nørskov , Adv. Catal. 2000, 31, 71.

[49] R. Kubo , A. Kawabata , S. Kobayashi , Annu. Rev. Mater. Sci. 1984, 14, 49.

[50] W. P. Halperin , Rev. Mod. Phys. 1986, 58, 533.

[51] X. Du , I. Skachko , A. Barker , E. Y. Andrei , Nat. Nanotechnol. 2008, 3, 491. 18685637 10.1038/nnano.2008.199

[52] T. Dürkop , B. M. Kim , M. S. Fuhrer , J. Phys.: Condens. Matter 2004, 16, R553.

[53] L. C. Campos , V. R. Manfrinato , J. D. Sanchez‐Yamagishi , J. Kong , P. Jarillo‐Herrero , Nano Lett. 2009, 9, 2600. 19527022 10.1021/nl900811r

[54] D. Geng , B. Wu , Y. Guo , B. Luo , Y. Xue , J. Chen , G. Yu , Y. Liu , J. Am. Chem. Soc. 2013, 135, 6431. 23586921 10.1021/ja402224h

[55] K. Nakada , M. Fujita , G. Dresselhaus , M. S. Dresselhaus , Phys. Rev. B 1996, 54, 17954. 10.1103/physrevb.54.17954 9985930

[56] Z. Zhu , Y. Lu , D. Qiao , S. Bai , T. Hu , L. Li , J. Zheng , J. Am. Chem. Soc. 2005, 127, 15698. 16277500 10.1021/ja053844x

[57] M. Bedewy , E. R. Meshot , M. J. Reinker , A. J. Hart , ACS Nano 2011, 5, 8974. 22023221 10.1021/nn203144f

[58] B. R. Eggen , M. I. Heggie , G. Jungnickel , C. D. Latham , R. Jones , P. R. Briddon , Science 1996, 272, 87.

[59] A. J. Bissette , S. P. Fletcher , Angew. Chem., Int. Ed. 2013, 52, 12800. 10.1002/anie.201303822 24127341

[60] P. Casino , L. Miguel‐Romero , A. Marina , Nat. Commun. 2014, 5, 3258. 24500224 10.1038/ncomms4258

[61] M. R. Miller , K. A. Miller , J. Bian , M. E. James , S. Zhang , M. J. Lynch , P. S. Callery , J. M. Hettick , A. Cockburn , J. Liu , C. Li , B. R. Crane , N. W. Charon , Nat. Microbiol. 2016, 1, 16134. 27670115 10.1038/nmicrobiol.2016.134 PMC5077173

[62] J. Tersoff , Nano Lett. 2015, 15, 6609. 26389697 10.1021/acs.nanolett.5b02386

[63] X. Yu , H. Wang , J. Lu , J. Zhao , J. Misuraca , P. Xiong , S. von Molnar , Nano Lett. 2012, 12, 5436. 22984828 10.1021/nl303323t

[64] Z. Wang , X. Kong , J. Zuo , Phys. Rev. Lett. 2003, 91, 185502. 14611289 10.1103/PhysRevLett.91.185502

[65] Y. Cui , X. Liang , J. Chai , Z. Cui , Q. Wang , W. He , X. Liu , Z. Liu , G. Cui , J. Feng , Adv. Sci. 2017, 4, 1700174. 10.1002/advs.201700174 PMC5700653 29201612

[66] Z. Jia , Y. Zeng , P. Tang , D. Gan , W. Xing , Y. Hou , K. Wang , C. Xie , X. Lu , Chem. Mater. 2019, 31, 5625.

[67] R. M. Jacobberger , B. Kiraly , M. Fortin‐Deschenes , P. L. Levesque , K. M. Mcelhinny , G. J. Brady , R. R. Delgado , S. S. Roy , A. Mannix , M. G. Lagally , Nat. Commun. 2015, 6, 8006. 26258594 10.1038/ncomms9006 PMC4918381

[68] R. M. Jacobberger , E. A. Murray , M. Fortin‐Deschenes , F. Goltl , W. A. Behn , Z. J. Krebs , P. L. Levesque , D. E. Savage , C. Smoot , M. G. Lagally , P. Desjardins , R. Martel , V. Brar , O. Moutanabbir , M. Mavrikakis , M. S. Arnold , Nanoscale 2019, 11, 4864. 30821309 10.1039/c9nr00713j

[69] Z. Yan , J. Lin , Z. Peng , Z. Sun , Y. Zhu , L. Li , C. Xiang , E. L. Samuel , C. Kittrell , J. Tour , ACS Nano 2012, 6, 9110. 22966902 10.1021/nn303352k

[70] L. Sun , L. Lin , J. Zhang , H. Wang , H. Peng , Z. Liu , Nano Res. 2017, 10, 355.

[71] Y. Liao , H. Jiang , N. Wei , P. Laiho , Q. Zhang , S. A. Khan , E. I. Kauppinen , J. Am. Chem. Soc. 2018, 140, 9797. 30049205 10.1021/jacs.8b05151 PMC6150687

[72] Y. Yao , C. Feng , J. Zhang , Z. Liu , Nano Lett. 2009, 9, 1673. 19284730 10.1021/nl900207v

[73] J. Liu , C. Wang , X. Tu , B. Liu , L. Chen , M. Zheng , C. Zhou , Nat. Commun. 2012, 3, 1199. 23149724 10.1038/ncomms2205

[74] B. Liu , J. Liu , X. Tu , J. Zhang , M. Zheng , C. Zhou , Nano Lett. 2013, 13, 4416. 23937554 10.1021/nl402259k

[75] Z. Zhu , N. Wei , W. Cheng , B. Shen , S. Sun , J. Gao , Q. Wen , R. Zhang , J. Xu , Y. Wang , F. Wei , Nat. Commun. 2019, 10, 4467. 31578325 10.1038/s41467-019-12519-5 PMC6775125

[76] A. J. Way , R. M. Jacobberger , M. S. Arnold , Nano Lett. 2018, 18, 898. 29382200 10.1021/acs.nanolett.7b04240

[77] A. J. Way , E. A. Murray , F. Golt , V. Saraswat , R. M. Jacobberger , M. Mavrikakis , M. S. Arnold , J. Phys. Chem. Lett. 2019, 10, 4266. 31287706 10.1021/acs.jpclett.9b01079

[78] R. T. K. Baker , Carbon 1989, 27, 315.

[79] A. M. Amin , E. Croiset , W. Epling , Int. J. Hydrogen Energy 2011, 36, 2904.

[80] D. Salari , A. Niaei , M. R. Shoja , R. Nabavi , Int. J. Chem. React. Eng. 2010, 8, 21.

[81] H. Wang , Y. Yuan , L. Wei , K. Goh , D. Yu , Y. Chen , Carbon 2015, 81, 1.

[82] M. He , L. Zhang , H. Jiang , H. Yang , F. Fossard , H. Cui , Z. Sun , J. B. Wagner , E. I. Kauppinen , A. Loiseau , Carbon 2016, 107, 865.

[83] M. He , H. Jiang , E. I. Kauppinen , J. Lehtonen , Nanoscale 2012, 4, 7394. 23085735 10.1039/c2nr32276e

[84] Q. Wen , R. Zhang , W. Qian , Y. Wang , P. Tan , J. Nie , F. Wei , Chem. Mater. 2010, 22, 1294.

[85] Q. Wen , W. Qian , J. Nie , A. Cao , G. Ning , Y. Wang , L. Hu , Q. Zhang , J. Huang , F. Wei , Adv. Mater. 2010, 22, 1867. 20512964 10.1002/adma.200902746

[86] J. Ma , D. Alfe , A. Michaelides , E. Wang , Phys. Rev. B 2009, 80, 033407.

[87] F. Banhart , J. Kotakoski , A. V. Krasheninnikov , ACS Nano 2011, 5, 26. 21090760 10.1021/nn102598m

[88] A. J. Stone , D. J. Wales , Chem. Phys. Lett. 1986, 128, 501.

[89] Q. Yuan , Z. Xu , B. I. Yakobson , F. Ding , Phys. Rev. Lett. 2012, 108.. 10.1103/PhysRevLett.108.245505 23004292

[90] L. Li , S. Reich , J. Robertson , Phys. Rev. B 2005, 72, 184109.

[91] S. Nie , J. M. Wofford , N. C. Bartelt , O. D. Dubon , K. F. McCarty , Phys. Rev. B 2011, 84, 155425.

[92] Y. Zhang , T. Gao , Y. Gao , S. Xie , Q. Ji , K. Yan , H. Peng , Z. Liu , ACS Nano 2011, 5, 4014. 21500831 10.1021/nn200573v

[93] X. Li , W. Cai , L. Colombo , R. S. Ruoff , Nano Lett. 2009, 9, 4268. 19711970 10.1021/nl902515k

[94] J. C. Charlier , T. W. Ebbesen , Ph. Lambin , Phys. Rev. B 1996, 53, 11108. 10.1103/physrevb.53.11108 9982683

[95] C. Garau , A. Frontera , D. Quinonero , A. Costa , P. Ballester , P. M. Deya , Chem. Phys. 2004, 303, 265.

[96] S. Zhang , X. Wang , F. Yao , M. He , D. Lin , H. Ma , Y. Sun , Q. Zhao , K. Liu , F. Ding , J. Zhang , Chem 2019, 5, 1182.

[97] M. M. Ugeda , I. Brihuega , F. Guinea , J. M. Gomez‐Rodriguez , Phys. Rev. Lett. 2010, 104, 096804. 20367003 10.1103/PhysRevLett.104.096804

[98] M. M. Ugeda , I. Brihuega , F. Hiebel , P. Mallet , J. Y. Veuillen , J. M. Gomez‐Rodriguez , F. Yndurain , Phys. Rev. B 2012, 85, 121402. 10.1103/PhysRevLett.109.196802 23215414

[99] X. Peng , R. Ahuja , Nano Lett. 2008, 8, 4464. 19368005 10.1021/nl802409q

[100] V. M. Pereira , J. M. B. L. dos Santos , A. H. Castro Neto , Phys. Rev. B 2008, 77, 115109.

[101] H. Rostami , E. Cappelluti , Phys. Rev. B 2017, 96, 054205.

[102] J. C. Charlier , Acc. Chem. Res. 2002, 35, 1063. 12484794 10.1021/ar010166k

[103] M. Ouyang , J. L. Huang , C. L. Cheung , C. M. Lieber , Science 2001, 291, 97. 11141554 10.1126/science.291.5501.97

[104] D. Y. Joh , L. H. Herman , S. Y. Ju , J. Kinder , M. A. Segal , J. N. Johnson , G. K. L. Chan , J. Park , Nano Lett. 2011, 11, 1. 20677774 10.1021/nl1012568

[105] W. Wu , J. Yue , X. Lin , D. Li , F. Zhu , X. Yin , J. Zhu , J. Wang , J. Zhang , Y. Chen , X. Wang , T. Li , Y. He , X. Dai , P. Liu , Y. Wei , J. Wang , W. Zhang , Y. Huang , L. Fan , L. Zhang , Q. Li , S. Fan , K. Jiang , Nano Res. 2015, 8, 2721.

[106] Q. Zhao , Z. Xu , Y. Hu , F. Ding , J. Zhang , Sci. Adv. 2016, 2, e1501729. 27386532 10.1126/sciadv.1501729 PMC4928984

